# Supplementary material for: Inferring single-cell copy number profiles through cross-cell segmentation of read counts
Source: BMC Genomics. 2024 Jan 2;25:25. doi: 10.1186/s12864-023-09901-5 (PMC10762977; doi:10.1186/s12864-023-09901-5)
Supplement: Supplementary file 1 — Additional file 1: Fig. S1. An example of segmentation results on simulated datasets. The top four subfigures depict LRC data of four tumor clones, and the bottom subfigure shows the learned latent sequence and segmentation results. Fig. S2. Analysis of the relationship between the ground truth average copy number and estimated baseline shift oof LRC signal. Data marked by blue, green and red are from tetraploid, triploid and diploid cells, respectively. Fig. S3. Comparison of cell ploidy estimation results between different methods. Average copy number (ACN) is calculated for each cell based on the inferred copy numberprofiles, and difference between the ground truth and inferred ACNs(denoted as ∆ACN) is analyzed for each method.Values of ∆ACN that are closer to 0 indicate better estimation results. Fig. S4. Analysis of the effect of bin size on copy number segments detection accuracy of DeepCNA. The values in {100kb, 200kb, 500kb, 1000kb} are tested for the bin size. Four performance metrics including mean of absolute distances (MAD), difference between the ground truth and inferred average copy numbers(denoted as ∆ACN), breakpoint distance and ratio between the number of inferred breakpoints and the number of real breakpoints(denoted as w)are calculated for comparison. Fig. S5. Analysis of the effect of latent dimensionality on breakpoint detection accuracy. The values in {1, 2, 3} are tested for the latent dimensionality d. Breakpoint distance and the metric ware calculated for comparison. Data points marked with an asteriskrepresent mean values. Fig. S6. Segmentation results of DeepCNA on three simulated datasets when the latent dimension is set to 2. The first dimension well captures most of the breakpoints, while sometimes underrepresents the breakpoints that may be shared by only a few cells, and these breakpoints are detectable from the second dimension. It is also observed that some breakpoints can be simultaneously detected from both dimensions. Fig. S7. [file 12864_2023_9901_MOESM1_ESM.pdf]

Inferring single-cell copy number profiles through cross-cell segmentation of read counts

Additional file 1

Furui Liu[1], Fangyuan Shi[1,2] and Zhenhua Yu[1,2*]
[1]School of Information Engineering and [2]Collaborative Innovation Center for Ningxia Big Data and Artificial Intelligence Co-founded by Ningxia Municipality and Ministry of Education, Ningxia University, Yinchuan 750021, China
*To whom correspondence should be addressed.
Contact: zhyu@nxu.edu.cn

Contents

1. Supplementary Figures 2
2. Supplementary Tables 18

1. Supplementary Figures

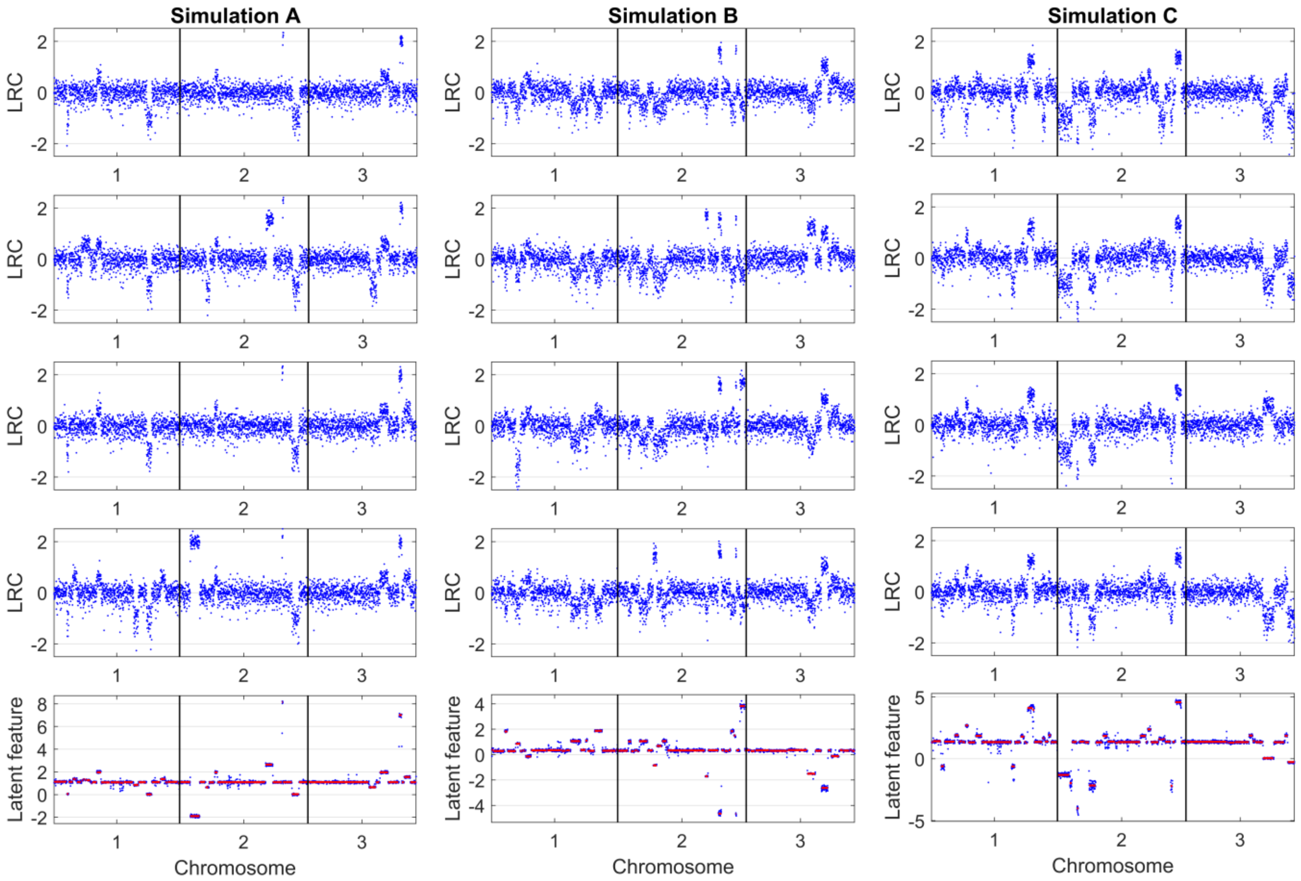

Fig. S1. An example of segmentation results on simulated datasets. The top four subfigures depict LRC data of four tumor clones, and the bottom subfigure shows the learned latent sequence and segmentation results.

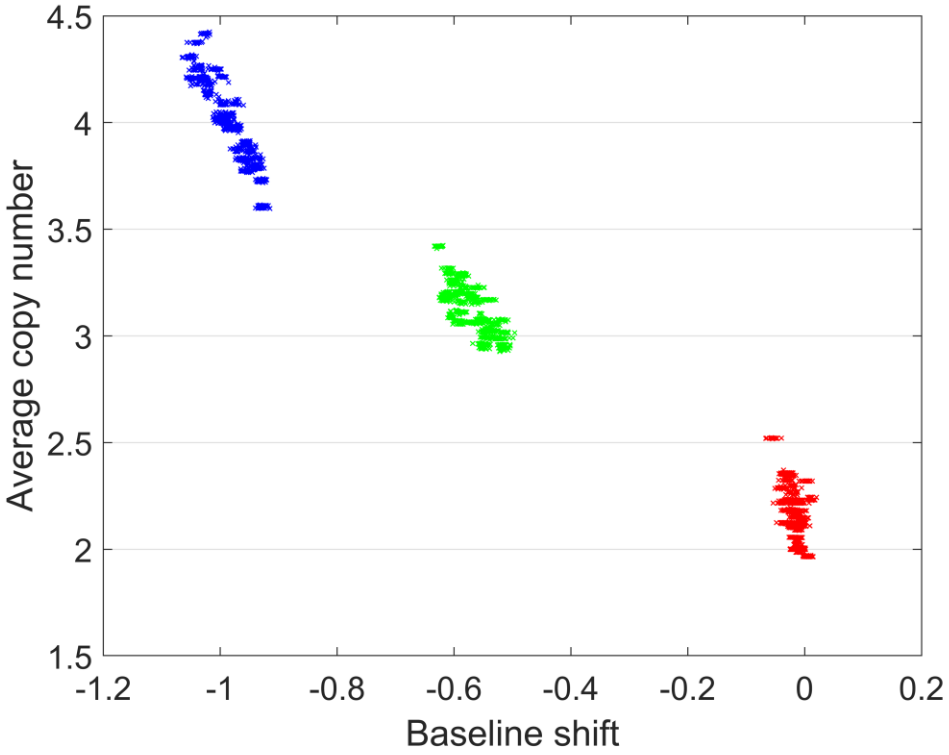

Fig. S2. Analysis of the relationship between the ground truth average copy number and estimated baseline shift *o* of LRC signal. Data marked by blue, green and red are from tetraploid, triploid and diploid cells, respectively.

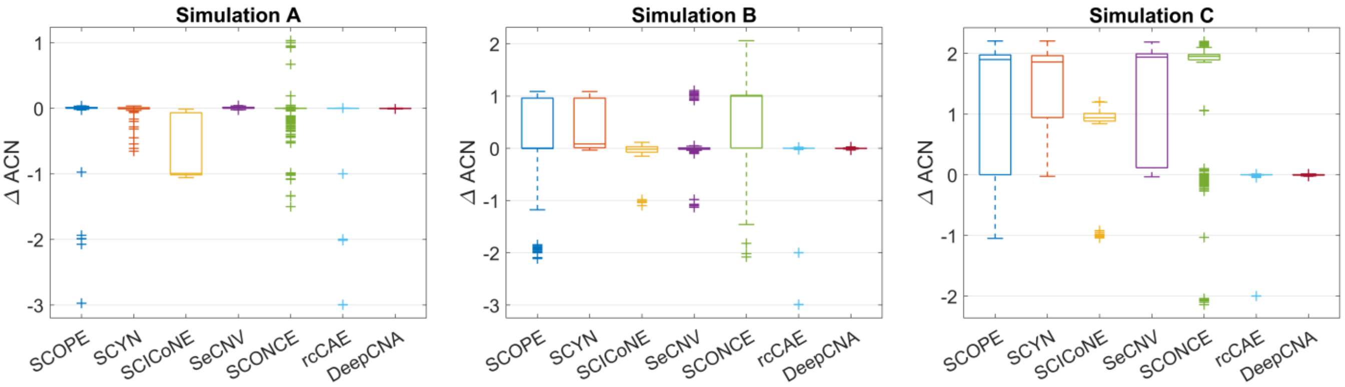

Fig. S3. Comparison of cell ploidy estimation results between different methods. Average copy number (ACN) is calculated for each cell based on the inferred copy number profiles, and difference between the ground truth and inferred ACNs (denoted as ΔACN) is analyzed for each method. Values of ΔACN that are closer to 0 indicate better estimation results.

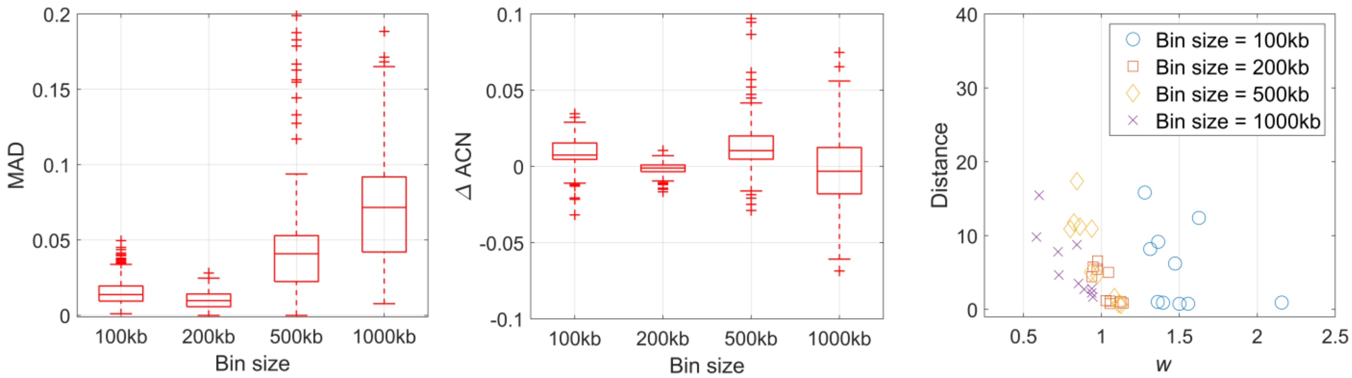

Fig. S4. Analysis of the effect of bin size on copy number segments detection accuracy of DeepCNA. The values in {100kb, 200kb, 500kb, 1000kb} are tested for the bin size. Four performance metrics including mean of absolute distances (MAD), difference between the ground truth and inferred average copy numbers (denoted as ΔACN), breakpoint distance and ratio between the number of inferred breakpoints and the number of real breakpoints (denoted as *w*) are calculated for comparison.

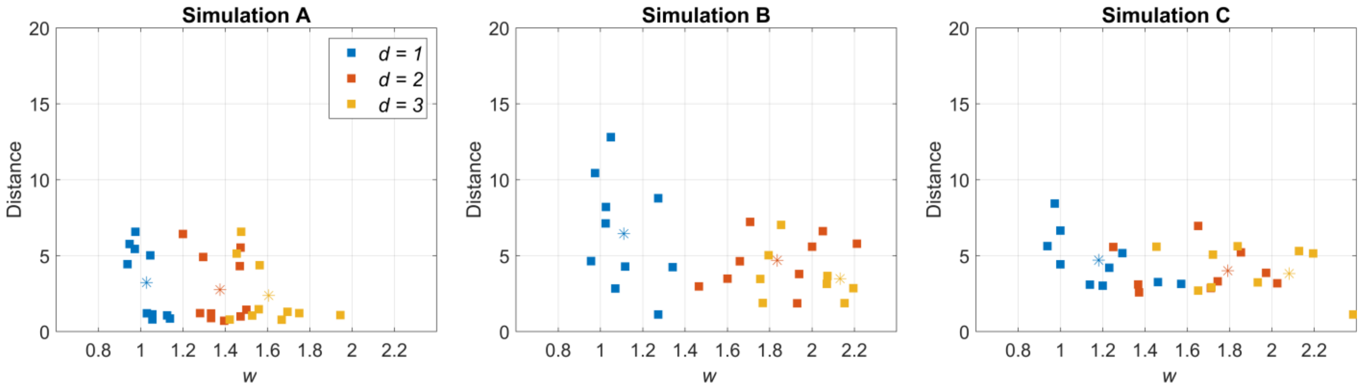

Fig. S5. Analysis of the effect of latent dimensionality on breakpoint detection accuracy. The values in {1, 2, 3} are tested for the latent dimensionality d. Breakpoint distance and the metric w are calculated for comparison. Data points marked with an asterisk represent mean values.

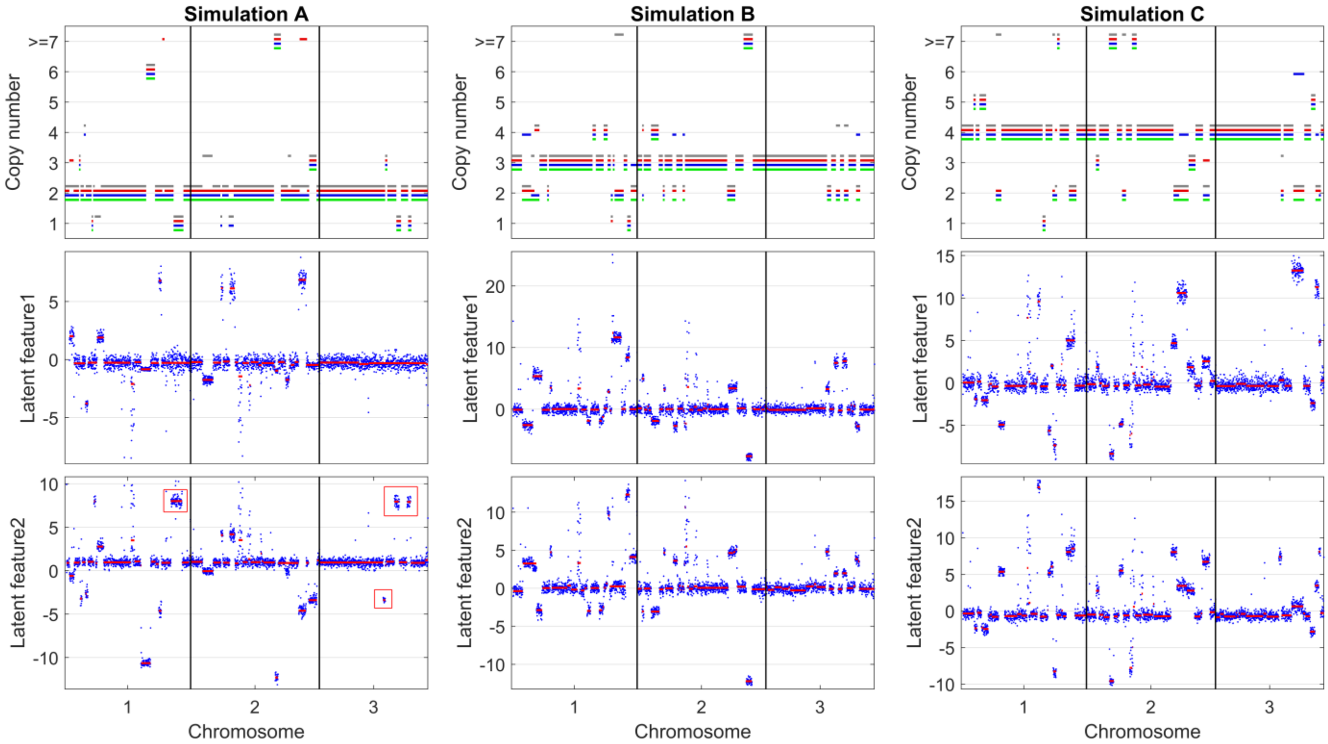

Fig. S6. Segmentation results of DeepCNA on three simulated datasets when the latent dimension is set to 2. The first dimension well captures most of the breakpoints, while sometimes underrepresents the breakpoints that may be shared by only a few cells, and these breakpoints are detectable from the second dimension. It is also observed that some breakpoints can be simultaneously detected from both dimensions.

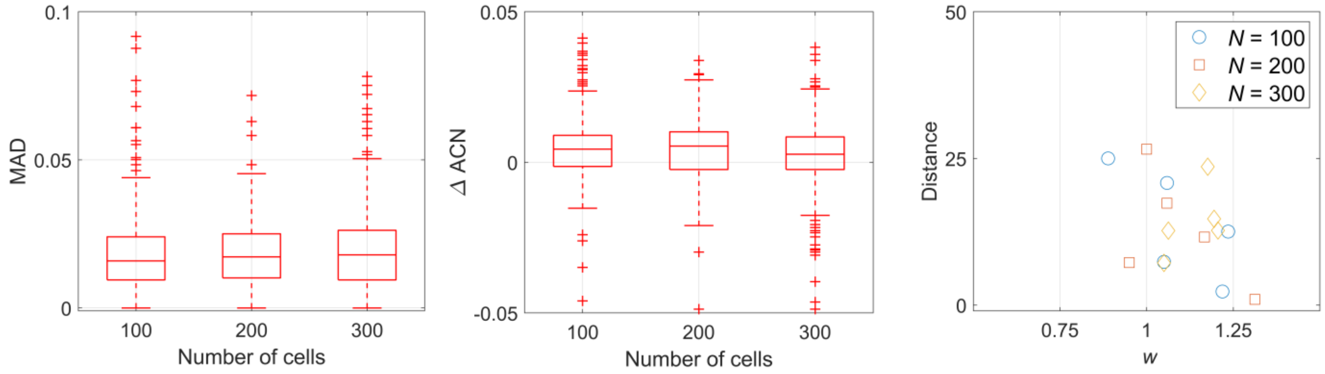

Fig. S7. Analysis of DeepCNA' performance on different-sized datasets. The numbers of cells in {100, 200, 300} are tested. Four performance metrics including mean of absolute distances (MAD), difference between the ground truth and inferred average copy numbers (denoted as ΔACN), breakpoint distance and ratio between the number of inferred breakpoints and the number of real breakpoints (denoted as *w*) are calculated for comparison.

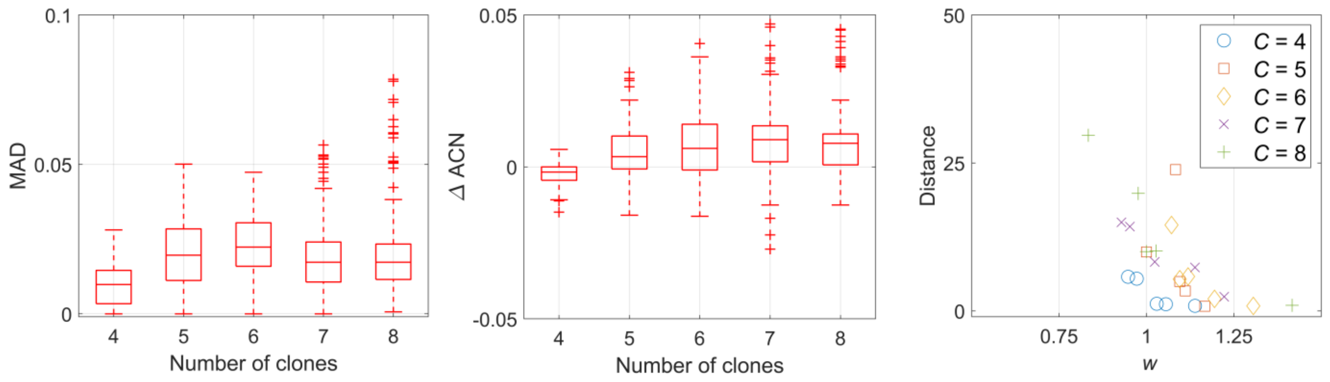

Fig. S8. Analysis of DeepCNA' performance on datasets with varying data heterogeneity. The numbers of clones changes from 4 to 8. Four performance metrics including mean of absolute distances (MAD), difference between the ground truth and inferred average copy numbers (denoted as ΔACN), breakpoint distance and ratio between the number of inferred breakpoints and the number of real breakpoints (denoted as *w*) are calculated for comparison. *C* denotes the number of clones.

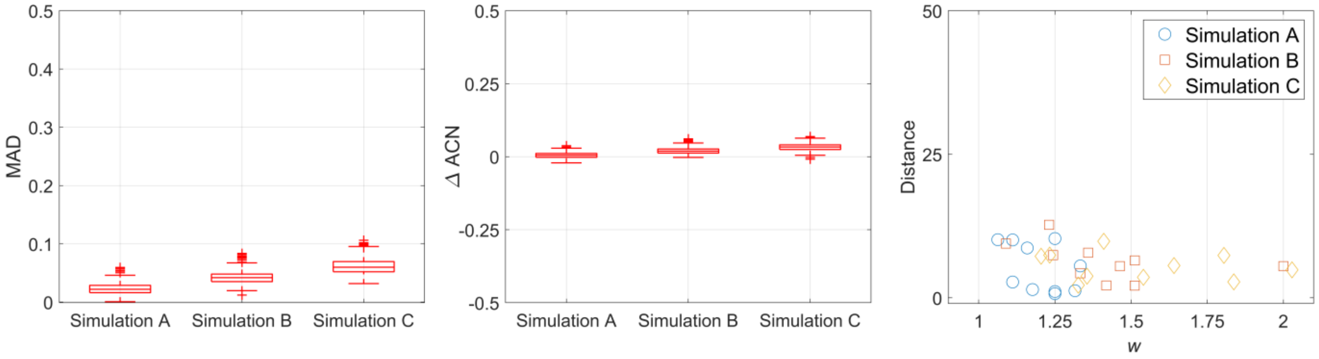

Fig. S9. Analysis of DeepCNA' performance on datasets with no normal cells mixed in the data. Four performance metrics including mean of absolute distances (MAD), difference between the ground truth and inferred average copy numbers (denoted as ΔACN), breakpoint distance and ratio between the number of inferred breakpoints and the number of real breakpoints (denoted as *w*) are calculated for comparison.

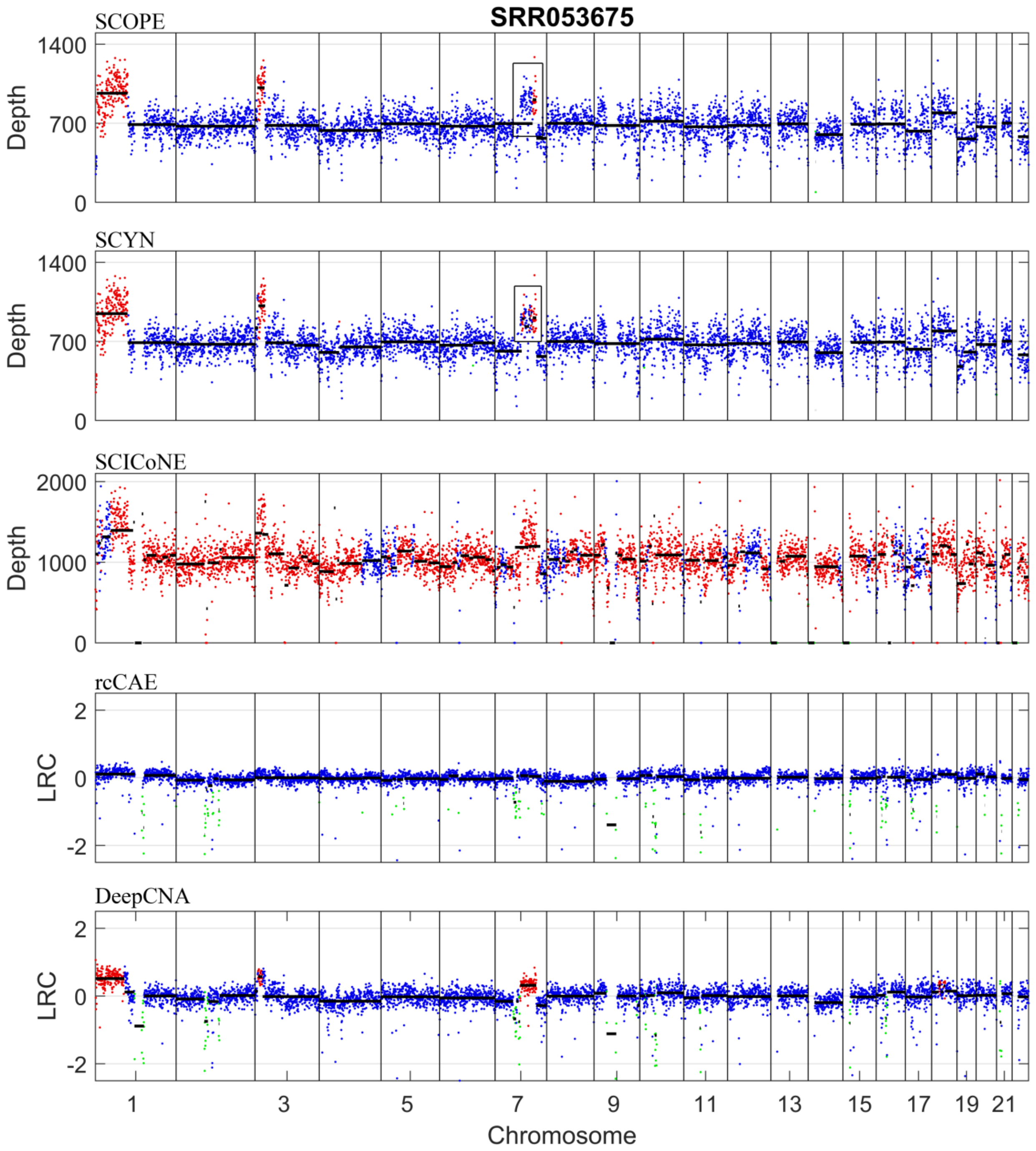

Fig. S10. Copy number estimation results on cell "SRR053675" from the breast cancer dataset. DeepCNA detects a copy number amplification on chromosome 7, while SCOPE, SCYN and rcCAE predict it as normal copy number, and their predictions deviate from the observed data. SCICoNE overestimates the cell ploidy. Copy number deletion, normal copy number and copy number amplification are marked by green, blue and red, respectively.

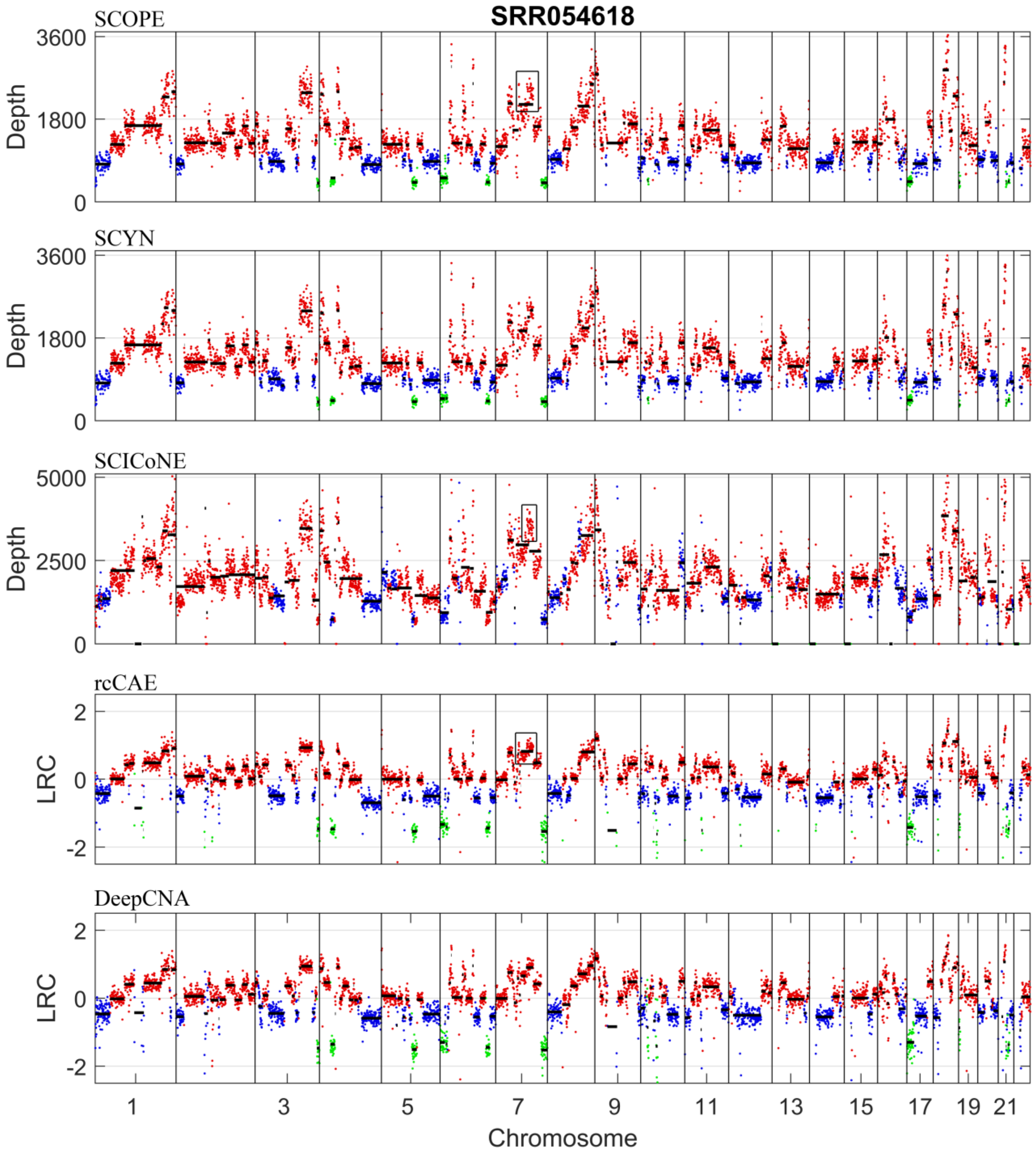

Fig. S11. Copy number estimation results on cell "SRR054618" from the breast cancer dataset. DeepCNA detects a small copy number segment on chromosome 7 that is missed by SCOPE, SCICoNE and rcCAE. Copy number deletion, normal copy number and copy number amplification are marked by green, blue and red, respectively.

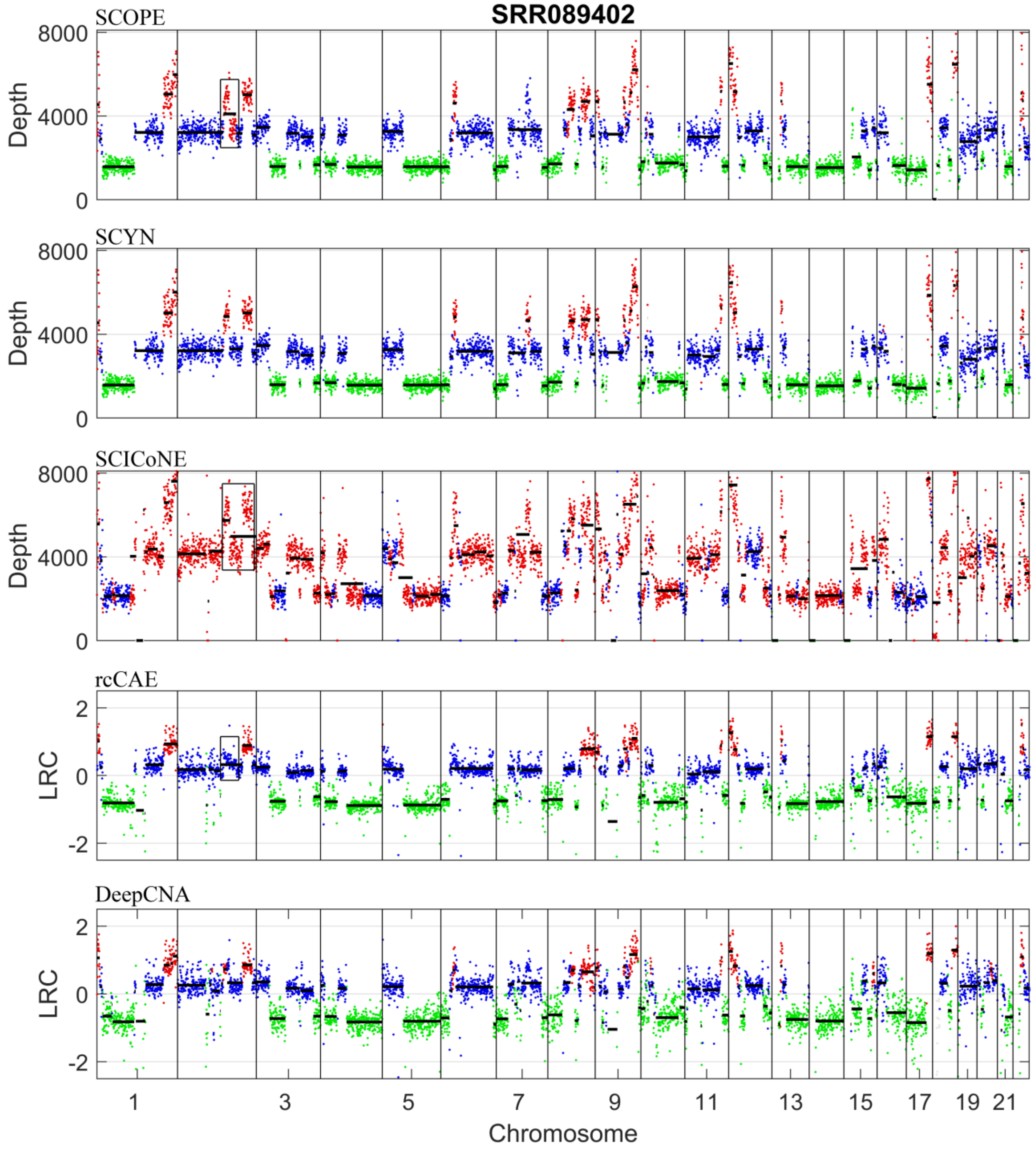

Fig. S12. Copy number estimation results on cell "SRR089402" from the breast cancer dataset. Existing methods except SCYN underestimate the number of breakpoints on chromosome 2q, thus yield biased predictions of the copy numbers. SCICoNE overestimates the cell ploidy. Copy number deletion, normal copy number and copy number amplification are marked by green, blue and red, respectively.

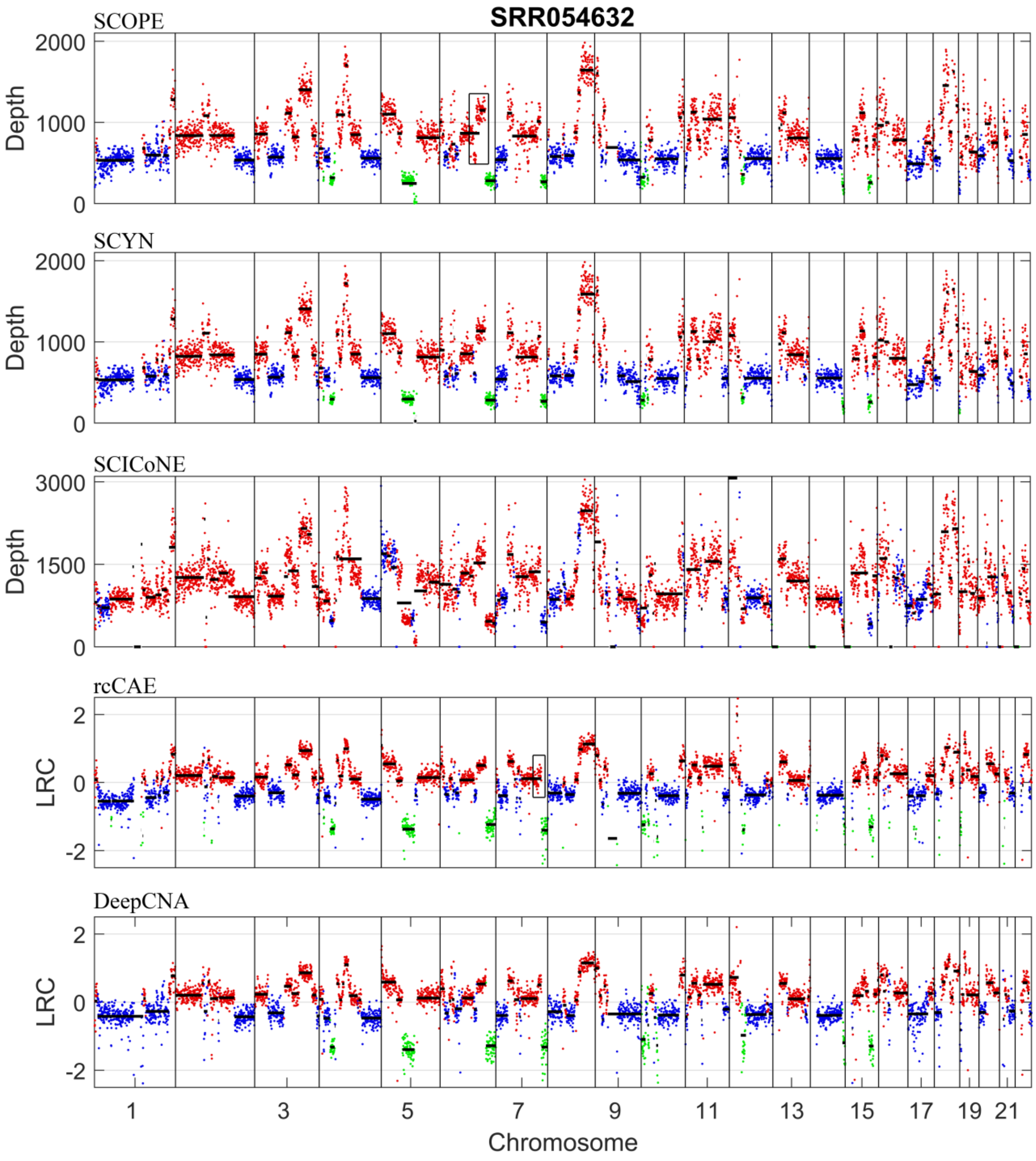

Fig. S13. Copy number estimation results on cell "SRR054632" from the breast cancer dataset. SCOPE and SCICoNE predict a small segment on chromosome 6 to copy number amplification. rcCAE fails to find a small segment on chromosome 7. Copy number deletion, normal copy number and copy number amplification are marked by green, blue and red, respectively.

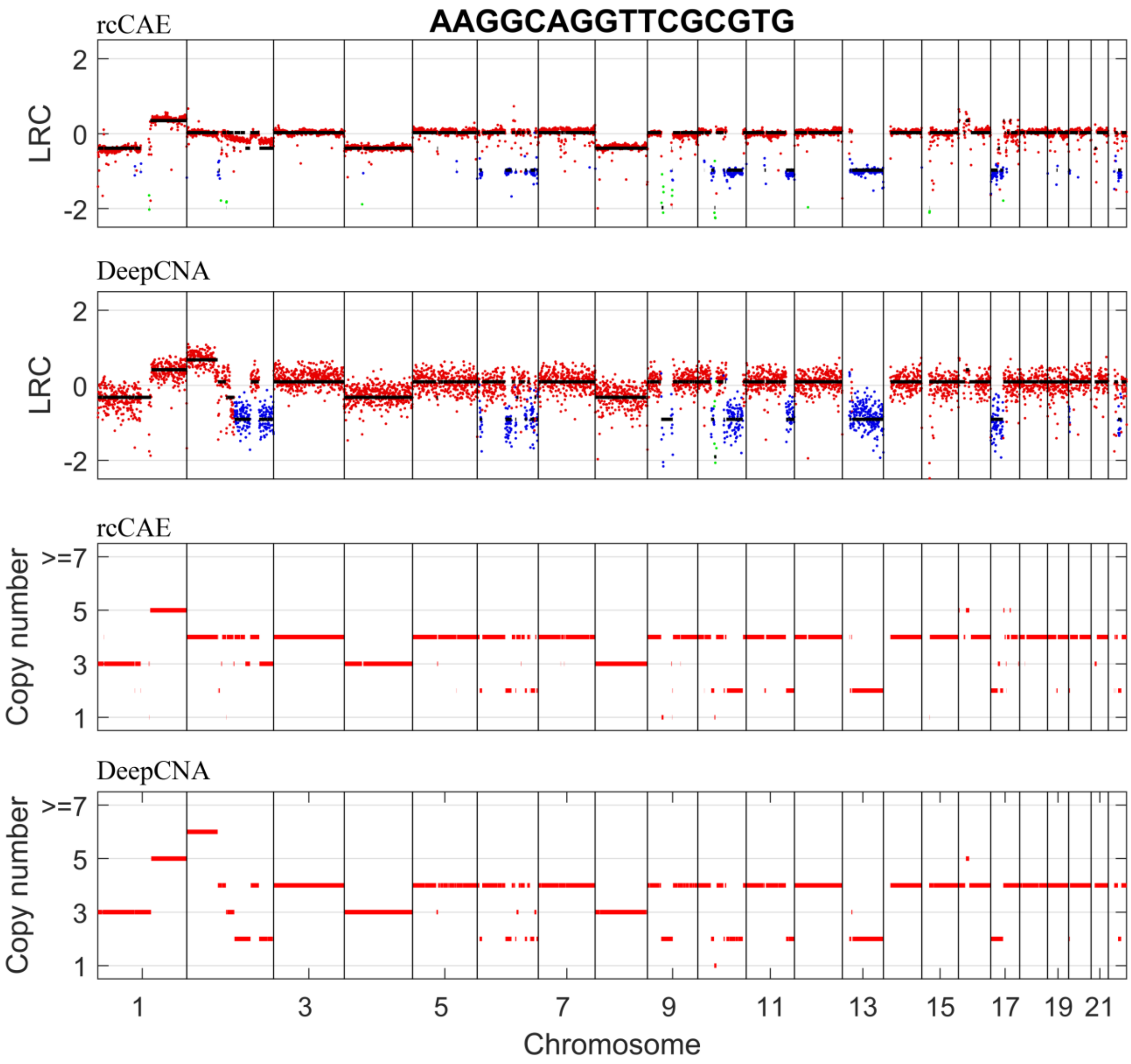

Fig. S14. Copy number estimation results on the cell with barcode "AAGGCAGGTTCGCGTG" from the 10X Genomics dataset. rcCAE' predictions on chromosome 2 probably deviate from the ground truth. Copy number deletion, normal copy number and copy number amplification are marked by green, blue and red, respectively.

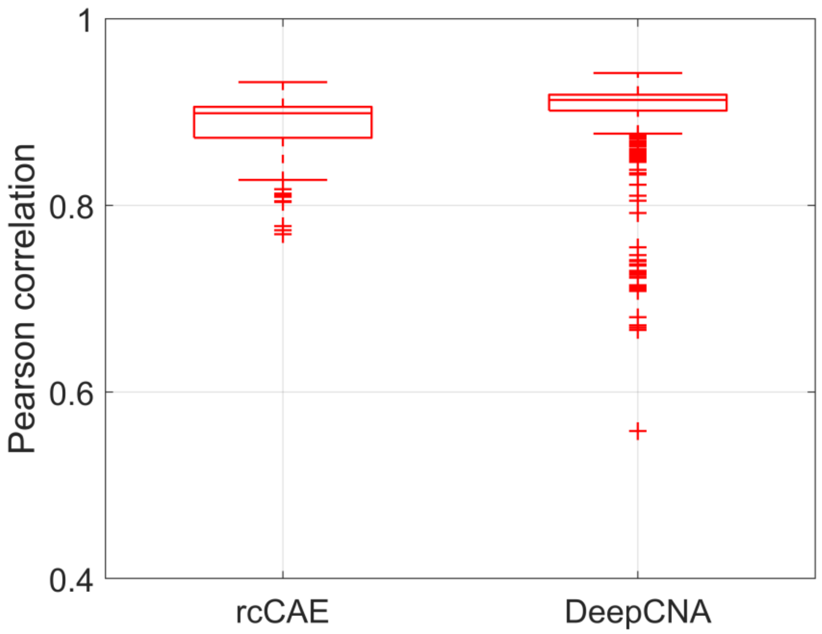

Fig. S15. Comparison of Pearson correlation coefficients on the 10X Genomics dataset. By using CHISEL results as the baseline, Pearson correlation coefficients are calculated based on copy numbers inferred by each method.

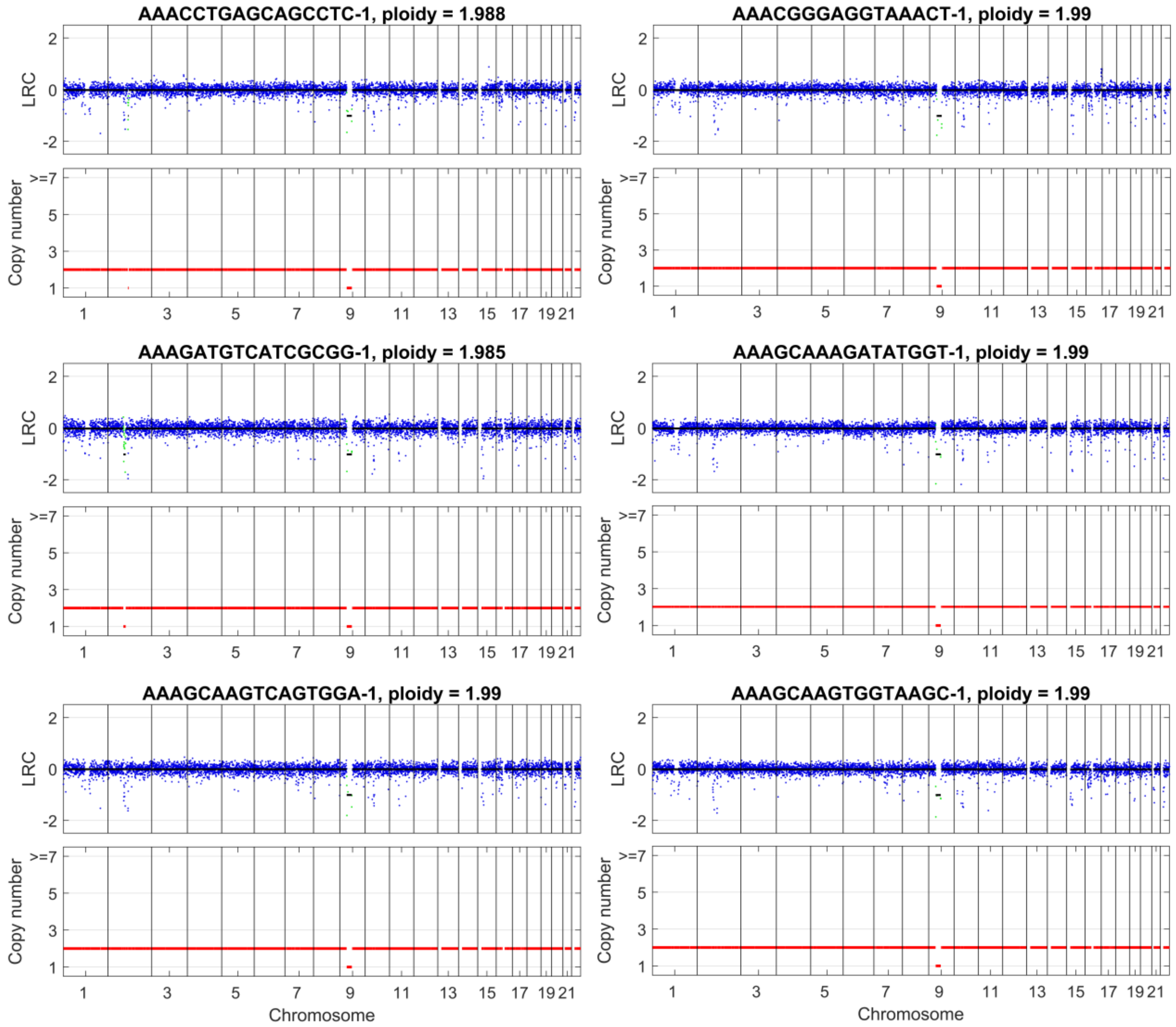

Fig. S16. An example of copy number estimation results of DeepCNA on the BJ Fibroblast Euploid Cell Line dataset. DeepCNA predicts all cells as diploidy.

2. Supplementary Tables

Table S1. The parameters used to run each method on simulated datasets.

**Methods**	**Specific parameters**
SCOPE	autochr = 3 in file “get_bam_bed.R”, resolution = 200 in file “run_scope.R”
SCYN	--seq paired-end --bin_len 200 --ref hg19 --reg .*[0-9].bam$ --mapq 40
SCICoNE	obtain read counts using BedTools, then use default parameters to call CNAs
SeCNV	-r hg19 -b 200000 -p *[0-9].bam
SCONCE	obtain read counts using BedTools, then use default parameters to call CNAs
rcCAE	prepInput: -r hg19.fa -m wgEncodeCrgMapabilityAlign36mer.bigWig -c 1,2,3 -s 200000<br>train.py: --epochs 200 --batch_size 64 --latent_dim 3 --seed 0
DeepCNA	prepInput: -r hg19.fa -m wgEncodeCrgMapabilityAlign36mer.bigWig -c 1,2,3 -s 200000<br>train.py: --epochs 500 --batch_size 256 --lr 0.0001 --latent_dim 1 --seed 0

Table S2. The runtime performance comparison results of all investigated methods. The results are obtained from simulated datasets with varying number of cells. All the experiments are conducted on a computational server with 64 CPU cores, 128 GB RAM and 1 GeForce RTX 2080 Ti GPU.

**Methods**	**Number of cells**	**Peak memory (MB)**	**Time (seconds)**
SCOPE	100	2714	3813
	200	3758	8245
	300	5103	14429
SCYN	100	2670	367
	200	2830	449
	300	2648	560
SCICoNE	100	723	149
	200	832	255
	300	941	343
SeCNV	100	1988	1408
	200	1988	1947
	300	1988	2474
SCONCE	100	121	16463
	200	122	27295
	300	122	44012
rcCAE	100	2677	69
	200	2694	116
	300	2703	177
DeepCNA	100	2553	65
	200	2558	95
	300	2566	124
